# The Influence of the Inertial Motor Unit Location (Lumbosacral vs. Thoracic Regions) on the External Load Registered During Badminton Matches

**DOI:** 10.3390/s25061910

**Published:** 2025-03-19

**Authors:** Juan García-López, José Pino-Ortega, Jaime Fernández-Fernández, José Vicente García-Tormo

**Affiliations:** 1 Faculty of Physical Activity and Sports Sciences, Universidad de León, 24071 León, Spain; juan.garcia@unileon.es (J.G.-L.); jvgart@unileon.es (J.V.G.-T.); 2 Department of Physical Activity and Sport, Faculty of Sport Sciences, University of Murcia, 30720 San Javier, Spain; josepinoortega@um.es

**Keywords:** training monitoring, racket sports, wearable sensors

## Abstract

The use of inertial motor units (IMUs) to monitor external training loads during training and competition has grown, particularly in racket sports like badminton. Previous studies highlighted the influence of sensor location on external load measurements, with the lumbosacral region identified as optimal. However, IMUs are often placed dorsally between the scapulae. This study examined the impact of IMU placement (lumbosacral vs. thoracic) on external load recordings during two simulated badminton matches. Sixteen junior international-level players (10 males, 6 females) participated in matches designed to replicate worst-case scenarios (2 × 35 min, 15 min rest). IMUs located on the lumbosacral joint (L) and thoracic area (T) recorded data combining Ultra-Wideband and acceleration technologies. The results showed higher total and sprint distances in T than L (1.0–3.6%, pη^2^ = 0.089–0.182). Small differences were noted for accelerations and decelerations (1.5%, pη^2^ = 0.057) with no significant differences in speed. Conversely, L showed higher values for total impacts and player load (34.6–49.8%, pη^2^ = 0.861–0.868). The findings reveal slight discrepancies in distance and speed based on placement but significant differences in impacts and player loads, warranting further investigation.

## 1. Introduction

The concepts of internal training loads and external training loads were defined at the beginning of the 21st century to distinguish the measurable aspects occurring internally or externally to the athlete [1]. The training/competition demands determine the external training loads (in terms of displacement, speed, acceleration, power, etc.), which elicit an internal psychophysiological response of the athlete (heart rate, lactatemia, subjective perception of effort, etc.) [1]. Therefore, monitoring external training loads has the potential to optimize athletic performance [2] by individualizing training loads [3] and enabling the replication of competition demands, which may also contribute to the prevention of sports injuries [4]. Although its effectiveness depends on the correct interpretation and application of the collected data.

Around 2010, the availability of technologies such as the Global Positioning System (GPS) enhanced the monitoring of external training loads in outdoor sports, including soccer [3], rugby [5], Australian football [6], and others. In recent years, the use of Ultra-Wideband technology (UWB), inertial motor units (IMUs), and their combination has grown significantly for monitoring external training loads during training and competition in indoor environments [2,7] as well as in striking sports [8,9]. It is necessary to remark that particularly racket sports such as padel, badminton, and tennis rank among the most widely practiced sports worldwide [9].

The aforementioned technologies are capable of measuring the total distance and cover distances in different speed zones, the number and intensity of the accelerations and decelerations, the frequency and intensity of the impacts, and the player load [9,10,11]. This last variable (i.e., player load) is derived from changes in acceleration data across the three axes of movement (i.e., anterior–posterior, medial–lateral, and vertical), and tries to provide an arbitrary unit representative of the total external load of training or competition [10].

In badminton, several studies monitored external load using UWB and IMUs [12,13,14]. One of them stated that males exhibited a higher number of accelerations relative to playing time and achieved greater maximum acceleration during matches compared to females, without differences in the rest of the analyzed variables [12]. Furthermore, another study observed that players who lost experienced a higher player load relative to game time than those who won [13]. However, the location of the sensors (i.e., UWB or IMUs) may influence the measurement of external training loads [14]. According to this last study, which compared sensor placement on the lumbosacral area, both wrists, and both thighs, the lumbosacral area was identified as the optimal location for these devices.

Nevertheless, there is currently no established gold standard for the sensor location, as the placement of the device varies across studies [15]. In most sports with changes in direction the sensor is located on the dorsal area (i.e., between the two scapulae) of the players’ body, possibly because it is easy to place a harness on that area [15]. This location and the elasticity of the harness can generate ’whipping’ movements, which are likely responsible for the extraneous accelerations [16] which could affect the monitoring of the external load. Additionally, this location may be less representative of the center of gravity’s movements than the lumbosacral location [16]. Therefore, the main purpose of the present study is to determine the influence of the IMU’s location (lumbosacral vs. thoracic areas) on the external load recordings during two simulated badminton matches.

## 2. Materials and Methods

### 2.1. Participants

Sixteen well-trained junior badminton players (10 males, 6 females, age: 16.2 ± 0.8 years, body mass: 63.5 ± 6.6 kg, height: 173.2 ± 6.3 cm) with international experience participated in the study. The inclusion criteria required that participants were not taking any medications throughout the study and had no musculoskeletal injuries in the three months prior to participation. The players and their parents were fully informed about the procedures and potential risks associated with participation, and written informed consent was obtained from both the players and their parents (for minors). The study was approved by the University Ethics Committee (ETICA-ULE-066-2023) and met the requirements of the Declaration of Helsinki.

### 2.2. Procedures

All measurements were taken from the players in the days leading up to an invitational singles badminton tournament held at a local badminton club in Oviedo, Spain. Upon arrival at the testing/competition venue (between 5:00 and 6:00 pm), which was well-ventilated and maintained at a constant temperature of approximately 18 °C with 40% relative humidity, the players underwent the assessments. Following a 10 min individual warm-up (e.g., jump rope, mobility exercises, and playing several points), participants engaged in two simulated badminton matches, each lasting 35 min, with a 15 min rest interval in between (total duration: 85 min), mimicking real competition scenarios [17]. Players were paired based on performance level (similar national rankings) and gender. The matches were friendly games held under Badminton World Federation rules, with the only exception that the game continued until the match time was completed, irrespective of the number of sets won. Each set was divided into two time intervals: from the beginning until the first player reached 11 points, and from that point until the end of the set. During both matches, each player completed between 5 and 6 sets (10–12 intervals). Pure water was provided on the sidelines for players to drink freely during the matches, at the end of each interval (60 s rest) or at the end of each set (3 min rest), as no additional breaks were scheduled.

### 2.3. System Calibration, IMUs Placement and Data Collection

**Figure 1:**
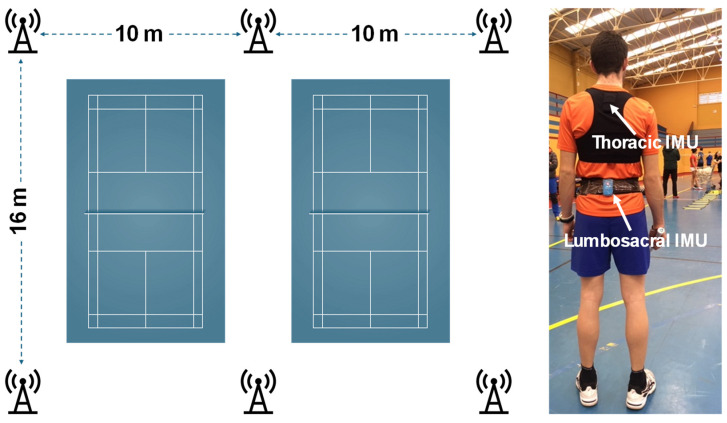
Placement of the six Ultra-Wideband antennas around the badminton courts. Positioning of the inertial motor units (IMUs) on the participating players: Thoracic IMU (second thoracic vertebra) and Lumbosacral IMU (L5-S1 intervertebral joint).

The matches were recorded on two parallel badminton courts, around which six Ultra-Wideband antennas were placed (Figure 1), in a similar way that previous studies did [12,18]. The antennas were arranged in a rectangle, spaced from 10 to 16 m apart, and mounted on tripods at a height of 3 m. Prior to the warm-up, all players were equipped with a special neoprene vest where the first IMU was attached at the interscapular level (Thoracic IMU), at approximately the level of the 2nd thoracic vertebra. The players and the researchers could choose from different sizes (e.g., small, medium, and large) to adjust the harness as securely as possible while ensuring it did not interfere with the typical movements of badminton. Additionally, the second IMU was placed at the lumbosacral level (Lumbosacral IMU), at approximately the L5-S1 intervertebral joint, over an elastic belt and secured with non-elastic adhesive tape. The goal was to stabilize the Lumbosacral IMU relative to the center of gravity and compare it with the most used configuration in sports (i.e., the Thoracic IMU).

The evaluation of the singles matches was carried out using inertial measurement units (IMU, WIMU PRO™, RealTrack Systems, Almería, Spain). These devices had their own internal microprocessor, 2 GB of flash memory, and a high-speed USB interface to record, store, and upload data. They were powered by an internal battery with a 4 h lifespan, had dimensions of 81 × 45 × 16 mm, and a total mass of 70 g [18]. Each device incorporated four accelerometers, a gyroscope, a magnetometer, and a GNSS and UWB chipset to capture data related to time-motion (outdoor: Global Navigation Satellite System, GNSS; indoor: Ultra-Wideband, UWB), specific actions/skills (accelerometer, gyroscope, magnetometer), and to connect with other sensors (Ant+, Bluetooth, Wi-Fi). The sensors operated at a sampling frequency of 18 Hz for UWB and 1000 Hz for the accelerometer, magnetometer, and gyroscope [12,19]. To ensure that both IMUs recorded the same time period, at the beginning and end of each match, two researchers manually started and stopped the recording of the two IMUs of each player at the signal of one of them. The two researchers manually recorded the time to later select and analyze the set intervals. At the end of the two consecutive matches, the IMUs were turned off and removed for analysis using the manufacturer’s software (S PRO™, RealTrack Systems, Almería). The software automatically generated all the variables described below as raw data, without applying any data cleaning.

The calibration of the Ultra-Wideband equipment (i.e., the global coordinate system calibration) was conducted according to protocols established in previous studies, with the intra-unit and inter-unit reliability of the WIMU PRO™ under indoor conditions already documented [12,18]. Briefly, the antennas were switched on sequentially, with the master antenna activated last. A 5 min auto-calibration protocol synchronized all antennas to a common clock. Afterward, at each position update (18 Hz), the master antenna sent a time synchronization signal. Then, the tracking devices were turned on, undergoing a 1 min recognition and communication process with the antennas. The accuracy of the system was tested, yielding a mean absolute error for the x-position of 5.2 ± 3.1 cm and for the y-position of 5.8 ± 2.3 cm [18]. Similarly, the accelerometer calibration followed the methodology outlined in prior research [19], which also evaluated intra- and inter-unit reliability, showing coefficients of variation below 3%, near-perfect inter-device correlations (r = 0.99–1.00), and very low day-to-day variations (r = 0.86–0.96).

The following variables were analyzed during each set interval (i.e., from the beginning until the first player reached 11 points, and from that point until the end of the set) and across the two simulated matches (i.e., the sum of all set intervals from both matches) similarly to previous studies [12,18,19,20,21,22]:

The variables obtained from the UWB, all of them derived from the player’s position on the court (x, y) and the time. Total distance (m): total distance covered. Explosive distance (m): distance covered with accelerations > 1.12 m·s^−2^. Accelerations: number of accelerations. Accelerations 0–2 m·s^−2^ (n): number of accelerations between 0 and 2 m·s^−2^. Accelerations 2–10 m·s^−2^ (n): number of accelerations between 2 and 10 m·s^−2^. Decelerations: number of decelerations. Decelerations 0–2 m·s^−2^ (n): number of decelerations between 0 and −2 m·s^−2^. Decelerations 2–10 m·s^−2^ (n): number of decelerations between −2 and −10 m·s^−2^. Maximum acceleration (m·s^−2^): maximal value of acceleration. Maximum deceleration (m·s^−2^): maximal value of deceleration. Average acceleration (m·s^−2^): mean value of acceleration. Average deceleration (m·s^−2^): mean value of deceleration. Maximum speed (km·h^−1^): maximal value of speed. Average speed (km·h^−1^): mean value of speed.

Variables obtained from accelerometers through sensor fusion of inertial device sensors (accelerometer, gyroscope, and magnetometer) along their three axes (vertical, anteroposterior, and lateral). Total impacts: number of impacts. Impacts 1.5–2.0 g: number of impacts between 1.5 and 2 g. Impacts 2.0–2.5 g: number of impacts between 2.0 and 2.5 g. Impacts 2.5–3.0 g: number of impacts between 2.5 and 3.0 g. Impacts 3.0–100.0 g: number of impacts between 3.0 and 100.0 g. Player Load (au): player load calculated as the square root of the sum of the squared acceleration rate changes at each moment of a training session across all movement axes (x, y, and z), and expressed in arbitrary units [22].

### 2.4. Statistical Analysis

The results are expressed as mean ± SD. When percentage differences were calculated, the following equation was used: Differences (%) = (maximum value − minimum value) × 100/minimum value. Data analysis was conducted using SPSS+ statistical software (v. 26.0, IBM Corp., Armonk, NY, USA). The Shapiro–Wilk test was applied to ensure a Gaussian distribution of all variables. The concordance between the IMUs measurements based on their location (i.e., Thoracic vs. Lumbosacral) was assessed using data from all set intervals, employing the intraclass correlation coefficient (ICC) and the Bland–Altman method, which included systematic bias ± random error [23]. The ICCs’ values were classified as “poor” (ICC < 0.5), “moderate” (0.5–0.75), “good” (0.75–0.9), and “excellent” (ICC > 0.9) [24]. Additionally, the 95% confidence interval (95% CI) was calculated. The effect of the IMU’s location on the studied variables (i.e., distance, accelerations, decelerations, player load, etc.) was analyzed using data from the two matches and performing a one-way repeated measures analysis of variance (ANOVA). Partial eta squared (η*_p_*^2^) was calculated as a measure of the effect size, with values classified as small (0.01–0.059), moderate (0.06–0.137), and large (>0.137) [25]. Newman–Keuls post hoc analysis was used to establish statistical differences between means. Values of *p* < 0.05 were considered statistically significant.

## 3. Results

Table 1 presents the results obtained with the two IMUs based on their location on the body, as well as their differences and correlations. The values for total and explosive distances were moderately to largely higher in the thoracic region compared to the lumbosacral region (1.0 and 3.6%, η*_p_*^2^ = 0.089 and 0.182, respectively). Small differences were observed in the number of accelerations and decelerations (1.5 and 1.5%, η*_p_*^2^ = 0.057 and 0.057), with small to negligible differences in maximum and average speeds (0.4 and –0.2%, η*_p_*^2^ = 0.011 and 0.000, respectively). Differences in the number of accelerations and decelerations were only significant between 2 and 10 m·s^−2^, but not between 0 and 2 m·s^−2^. Conversely, values for total impacts and player load were higher in the lumbosacral region compared to the thoracic region (49.8 and 34.6%, η*_p_*^2^ = 0.861 and 0.868, respectively). The number of impacts was consistently greater in the lumbosacral region, regardless of their magnitude (differences between 47.6 and 51.0% and η*_p_*^2^ between 0.661 and 0.908). In all the aforementioned variables, the differences were very large.

**Figure 2:**
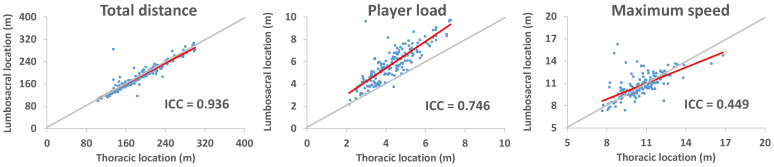
Graphical representation of the intraclass correlation coefficients (ICCs) for the total distance (excellent), player load (good), and maximum speed (poor). The red line represents the correlation between the variables, while the gray line represents the expected correlation.

The ICC values were statistically significant (Table 1); however, they ranged from poor to good for maximum acceleration and deceleration, average deceleration, maximum speed, number of impacts between 1.5 and 2.0 g, and player load. Figure 2 shows the ICCs for the total distance (excellent), player load (good), and maximum speed (poor).

**Figure 3:**
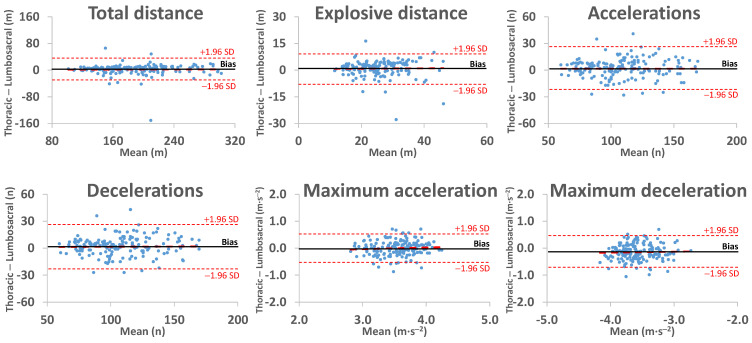

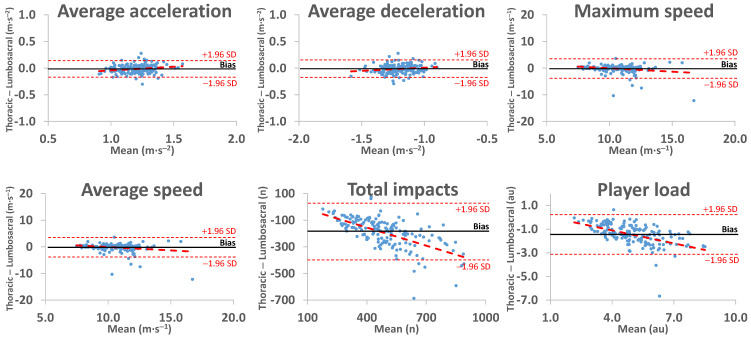
Bland–Altman plots of the differences between the measurements according to the IMU’s location (Thoracic and Lumbosacral). The short-dashed red lines represent the upper and lower 95% limits of agreement, the long-dashed red line represents the trend of the differences, and the solid black line represents the bias.

Figure 3 shows that the differences between the two locations were close to zero for most of the analyzed variables. Nevertheless, total impacts and player load were lower in the thoracic region compared to the lumbosacral region, with these differences increasing as the recorded values rose.

## 4. Discussion

The main finding of this study was that the IMU’s location (lumbosacral vs. thoracic areas) had little impact on the recorded distances and velocities, as well as on the number of accelerations and decelerations (i.e., values between 1 and 4% higher in the thoracic region compared to the lumbosacral one) during badminton simulated games. However, this placement significantly affected the magnitude of accelerations, the number of impacts, and, consequently, the player load (i.e., values over 30% higher in the lumbosacral region compared to the thoracic one). Additionally, the IMU’s location affected the agreement between measurements (i.e., ICC) and their consistency (i.e., Bland–Altman).

The greater total and explosive distances (~1–4%) recorded in the thoracic region (Table 1) can be attributed to the characteristic lunging movements, which account for approximately 14–20% of all movements in badminton [26,27]. During a lunge, which is a closed-chain movement involving triple flexion (hip, knee, and ankle) followed by triple extension in the dominant limb (racket side), the thorax moves more than the lumbosacral region due to hip and trunk flexion. Moreover, the lunge is one of the badminton movements that generates the highest levels of acceleration [27]. This, combined with the elasticity of the harness (i.e., “whipping” movements responsible for extraneous accelerations), likely explains why the number of recorded accelerations and decelerations was approximately 1.5% higher in the thoracic area than in the lumbosacral region [17].

Contrary to the previously mentioned differences in the variables obtained from the UWB, the variables derived from the accelerometers (i.e., the number of impacts and player load) were higher in the lumbosacral region than in the thoracic region (Table 1, ranging from 34.6% to 49.8%). This could be due to the following:

1. Player load is typically calculated based on impacts (i.e., accelerometer data) rather than accelerations derived from the player’s position (i.e., local positioning system data). This approach enables assessment using low-cost devices instead of expensive local positioning systems [28]. However, in the present study, this method led to the differences observed in the number of impacts also being reflected in the player load.

2. As a consequence of the above, a higher number of recorded impacts in the lumbosacral region than in the thoracic region was expected. As demonstrated in other activities such as running, a shock attenuation effect occurs from the anatomical regions closest to the ground up to the head [29]. The differences observed in the present study were more than twice those reported in a previous study (15.7 ± 9.7%), which compared the same locations at running speeds between 7 and 16 km/h [30]. This could be explained by the fact that badminton movements, due to greater joint flexion compared to running (e.g., lunges), resulted in increased shock attenuation [26,27].

3. The harness and its fit to the badminton player could compromise the reliability of the impact recording in the thoracic region. The present study showed that differences in the recorded number of impacts and player load increased as the values of these variables increased (Figure 3). Similarly, we encountered difficulties in comparing the number of impacts above 3 G (Table 1, 1040.9 ± 231.9 impacts in the thoracic region and 1550.0 ± 448.5 in the lumbosacral region) with studies that counted impacts above 4 G (1342 impacts in the thoracic region during two badminton matches) [27].

Thus, the agreement between recordings in the lumbosacral and thoracic regions for some variables (e.g., player load, acceleration and deceleration, and number of impacts) was compromised (Table 1 and Figure 2). This could be partially explained by the harness movements (i.e., “whipping” movements), as well as by the specific movements performed by badminton players (i.e., anterior–posterior and medio–lateral displacements, which may cause differences between the movements of the center of gravity and the thorax) [26,27]. In fact, when the harness is placed on the thorax during running, the reliability of anterior–posterior and medio–lateral movement recordings was lower compared to the vertical axis and the resultant measurement of all three axes [30]. Additionally, as mentioned before, the sensor placed in the lumbosacral region will record more accelerations than in the thoracic region due to greater impact attenuation in the latter. Future studies should assess this reliability during badminton-specific movements, comparing IMU placement in the lumbosacral and thoracic regions.

Considering all the aforementioned aspects, the kinematic variables obtained from local positioning systems (e.g., UWB) could be registered using sensors placed in either the lumbosacral or thoracic regions, as the observed differences were small and not dependent on the recorded magnitude (Figure 3). However, if the goal is to analyze the number and magnitude of impacts, as well as player load, it is crucial to ensure that the device is securely fixed in the thoracic region, as the recordings cannot be directly compared to those obtained in the lumbosacral region (Table 1, Figure 2 and Figure 3). In other words, from a practical perspective, the sensor placement would not be relevant for variables sensitive to sex, such as the number or magnitude of accelerations and decelerations [12]. However, it would be highly relevant to obtaining the player load, which was sensitive to the players’ level of practice [14]. Securely fixing the device in the thoracic region is not a simple task, as stabilizing the sensor in the lumbar region (i.e., L5-S1) is easier than in the thoracic region (i.e., T2), due to the latter having greater mobility and more soft tissue.

In the present study, the player load was calculated as proposed by the IMU manufacturer (i.e., RealTrack Systems). However, numerous accelerometry-based workload indexes exist across the different manufacturers. Therefore, a standardized index is necessary to enable comparisons between studies [22].

The main limitations of this study were as follows: (a) Not using an alternative system as a gold standard (e.g., video analysis tracking) to compare the player positioning recordings obtained from the lumbosacral and thoracic regions; (b) Not evaluating the reliability of the recordings obtained in both regions, which would have required standardizing the players’ movements (i.e., in a different context than match simulation); (c) The fact that the intraclass correlation coefficients and Bland–Altman plots were obtained from the intervals recorded for the same player (i.e., 10–12 match intervals per player) rather than from the total match could cause autocorrelation, which may overestimate regression statistics [31]. However, this approach was necessary to achieve a sufficient sample size for the calculation of ICCs [32]; (d) The sample size was relatively small (16 badminton players) but larger than the 10–12 participants used in previous studies on optimal wearable sensor placement [14,16]. Additionally, the effect size was calculated, and repeated intra-individual measurements to ensure that 16 participants provided sufficient inter-individual variability to support robust conclusions; (e) The Lumbosacral IMU was secured more tightly (i.e., with inelastic tape) than the thoracic IMU (i.e., with a neoprene harness), which could result in a combined effect of IMU location (lumbosacral vs. thoracic) and harness elasticity (i.e., inelastic tape vs. neoprene harness). Although the previous limitations should be considered in future studies, they did not affect the main conclusions of the present study.

## 5. Conclusions

The placement of the IMUs (lumbosacral vs. thoracic regions) has a relatively low influence (<4%) on measuring displacements during badminton matches, allowing for comparisons of results obtained from different players and studies regardless of device placement. However, its influence is significant when analyzing impacts and player load, as the results obtained from different placements are not interchangeable. Using the metrics from a sensor placed in a predetermined location can be useful for guiding badminton players’ training and recovery. Future studies should compare the reliability of these recordings based on sensor location during badminton-specific movements.

## Figures and Tables

**Table 1:** Results of the variables collected using the IMU’s location at the Thoracic (T) and Lumbosacral (L) body regions. Differences (F and η*_p_*^2^) and correlations (ICC) between the two locations.

	IMU’s LOCATION		Differences			Correlations	
	Thoracic (T)	Lumbosacral (L)	F	p	η*_p_*^2^	ICC	95% CI
Total Distance (m)	2202.2 ± 141.8	2179.4 ± 118.5	1.469	0.244	0.089	0.936	0.915–0.951
Explosive Distance (m)	290.2 ± 44.6	280.1 ± 36.3	3.340	0.088	0.182	0.836	0.787–0.874
Accelerations (n)	1287.8 ± 97.4	1268.7 ± 93.7	0.909	0.355	0.057	0.885	0.850–0.913
Accelerations 0–2 m·s^−2^ (n)	1062.8 ± 125.8	1059.3 ± 120.3	0.047	0.832	0.003	0.894	0.861–0.919
Accelerations 2–10 m·s^−2^ (n)	224.9 ± 50.0	209.4 ± 42.5 *	6.953	0.019	0.317	0.795	0.736–0.842
Decelerations (n)	1286.4 ± 98.2	1267.4 ± 93.7	0.908	0.356	0.057	0.885	0.850–0.913
Decelerations 0–2 m·s^−2^ (n)	1047.9 ± 118.7	1063.1 ± 112.1	0.853	0.370	0.054	0.887	0.852–0.914
Decelerations 2–10 m·s^−2^ (n)	238.5 ± 49.2	204.3 ± 31.1 *	30.475	0.000	0.670	0.792	0.732–0.840
Maximum acceleration (m·s^−2^)	3.79 ± 0.35	3.76 ± 0.33	1.475	0.243	0.090	0.735	0.662–0.794
Maximum deceleration (m·s^−2^)	−3.90 ± 0.29	–3.73 ± 0.27 *	29.440	0.000	0.662	0.605	0.506–0.688
Average acceleration (m·s^−2^)	1.18 ± 0.12	1.19 ± 0.10	4.071	0.062	0.213	0.828	0.776–0.868
Average deceleration (m·s^−2^)	–1.19 ± 0.12	–1.18 ± 0.09	2.061	0.172	0.121	0.800	0.742–0.846
Maximum speed (km·h^−1^)	13.8 ± 3.6	13.7 ± 2.7	0.003	0.960	0.000	0.449	0.327–0.556
Average speed (km·h^−1^)	3.2 ± 0.2	3.2 ± 0.2	0.170	0.686	0.011	0.903	0.872–0.926
Total Impacts (n)	4485.4 ± 820.0	6720.1 ± 1348.9 *	93.214	0.000	0.861	0.768	0.702–0.821
Impacts 1.5–2.0 g (n)	1881.1 ± 339.0	2840.2 ± 372.9 *	148.149	0.000	0.908	0.749	0.679–0.806
Impacts 2.0–2.5 g (n)	999.2 ± 201.5	1496.9 ± 334.1 *	90.146	0.000	0.857	0.726	0.650–0.787
Impacts 2.5–3.0 g (n)	564.2 ± 151.3	833.0 ± 243.2 *	53.817	0.000	0.782	0.708	0.629–0.773
Impacts 3.0–100.0 g (n)	1040.9 ± 231.9	1550.0 ± 448.5 *	29.187	0.000	0.661	0.627	0.532–0.707
Player Load (au)	50.0 ± 5.1	67.3 ± 8.6 *	98.518	0.000	0.868	0.803	0.746–0.849

Partial eta squared values (η*_p_*^2^) were classified as small (0.01–0.059), moderate (0.060–0.137), and large (>0.137). Intraclass coefficient of correlation (ICC) and 95% confidence interval (95% CI). * Significant differences between the two locations (*p* < 0.05). Abbreviations used: meter (m), number (n), meters per second squared (m·s^−2^), kilometers per hour (km·h^−1^), and arbitrary units (au).

## Data Availability

The raw data supporting the conclusions of this article will be made available by consulting the Supplementary Materials.

## Supplementary Materials

The following supporting information can be downloaded at: https://www.mdpi.com/article/10.3390/s25061910/s1. The raw data recorded for each participant (Table 1, Figure 2 and Figure 3).

## References

[1] Impellizzeri F.M., Marcora S.M., Coutts A.J. (2019). Internal and External Training Load: 15 Years On. Int. J. Sports Physiol. Perform..

[2] Gamonales J.M., Hernández-Beltrán V., Escudero-Tena A., Ibáñez S.I. (2023). Analysis of the External and Internal Load in Professional Basketball Players. Sports.

[3] Silva H., Nakamura F.Y., Castellano J., Marcelino R. (2023). Training Load Within a Soccer Microcycle Week—A Systematic Review. Strength Cond. J..

[4] Harper D.J., Carling C., Kiely J. (2019). High-Intensity Acceleration and Deceleration Demands in Elite Team Sports Competitive Match Play: A Systematic Review and Meta-Analysis of Observational Studies. Sports Med..

[5] Bradley E., Roberts J., Archer D. (2024). Determining female-specific high-intensity activity GPS thresholds in women’s rugby union: Use of current use of male-derived absolute speed thresholds underestimates true levels. Eur. J. Sport Sci..

[6] Rennie M.J., Kelly S.J., Bush S., Spurrs R.W., Sheehan W.B., Watsford M.L. (2022). Phases of Match-Play in Professional Australian Football: Positional Demands and Match-Related Fatigue. Sensors.

[7] Villarejo-García D.H., Moreno-Villanueva A., Soler-López A., Reche-Soto P., Pino-Ortega J. (2023). Use, Validity and Reliability of Inertial Movement Units in Volleyball: Systematic Review of the Scientific Literature. Sensors.

[8] Whiteside D., Cant O., Connolly M., Reid M. (2017). Monitoring Hitting Load in Tennis Using Inertial Sensors and Machine Learning. Int. J. Sports Physiol. Perform..

[9] Brich Q., Casals M., Crespo M., Reid M., Baiget E. (2024). Quantifying Hitting Load in Racket Sports: A Scoping Review of Key Technologies. Int. J. Sports Physiol. Perform..

[10] Bredt S.G.T., Chagas M.H., Peixoto G.H., Menzel H.J., Pereira de Andrade A.G. (2020). Understanding Player Load: Meanings and Limitations. J. Hum. Kinet..

[11] Teixeira J.E., Forte P., Ferraz R., Leal M., Ribeiro J., Silva A.J., Barbosa T.M., Monteiro A.M. (2021). Monitoring Accumulated Training and Match Load in Football: A Systematic Review. Int. J. Environ. Res. Public Health.

[12] Rojas-Valverde D., Gómez-Carmona C.D., Fernández-Fernández J., García-López J., García-Tormo V., Cabello-Manrique D., Pino-Ortega J. (2020). Identification of games and sex-related activity profile in junior international badminton. Int. J. Perform. Anal. Sport.

[13] Fu Y., Liu Y., Chen X., Li Y., Li B., Wang X., Shu Y., Shang L. (2021). Comparison of Energy Contributions and Workloads in Male and Female Badminton Players During Games Versus Repetitive Practices. Front. Physiol..

[14] Liu T.-H., Chen W.-H., Shih Y., Lin Y.-C. (2021). Better position for the wearable sensor to monitor badminton sport training loads. Sports Biomech..

[15] Alanen A.M., Räisänen A.M., Benson L.C., Pasanen K. (2021). The use of inertial measurement units for analyzing change of direction movement in sports: A scoping review. Int. J. Sports Sci. Coach..

[16] Edwards S., White S., Humphreys S., Robergs R., O’Dwyer N. (2019). Caution using data from triaxial accelerometers housed in player tracking units during running. J. Sports Sci..

[17] Phomsoupha M., Laffaye G. (2015). The science of badminton: Game characteristics, anthropometry, physiology, visual fitness and biomechanics. Sports Med..

[18] Bastida-Castillo A., Gómez-Carmona C.D., De la Cruz-Sánchez E., Reche-Royo X., Ibáñez S.J., Pino Ortega J. (2019). Accuracy and Inter-Unit Reliability of Ultra-Wide-Band Tracking System in Indoor Exercise. Appl. Sci..

[19] Gómez-Carmona C.D., Bastida-Castillo A., García-Rubio J., Ibáñez S.J., Pino-Ortega J. (2019). Static and dynamic reliability of WIMU PRO™ accelerometers according to anatomical placement. Proc. IMechE Part P J. Sports Eng. Technol..

[20] Oliva-Lozano J.M., Gómez-Carmona C.D., Fortes V., Pino-Ortega J. (2022). Effect of training day, match, and length of the microcycle on workload periodization in professional soccer players: A full-season study. Biol. Sport.

[21] Miralles-Iborra A., Elvira J.L.L., Del Coso J., Hernandez-Sanchez S., Pino-Ortega J., Moreno-Pérez V. (2024). Influence of a football match on landing biomechanics and jump performance in female football players. Scand. J. Med. Sci. Sports.

[22] Gómez-Carmona C.D., Bastida-Castillo A., Ibáñez S.J., Pino-Ortega J. (2020). Accelerometry as a method for external workload monitoring in invasion team sports. A systematic review. PLoS ONE.

[23] Ogueta-Alday A., Morante J.C., Rodríguez-Marroyo J.A., García-López J. (2013). Validation of a new method to measure contact and flight times during treadmill running. J. Strength Cond. Res..

[24] Koo T.K., Li M.Y. (2016). A Guideline of Selecting and Reporting Intraclass Correlation Coefficients for Reliability Research. J. Chiropr. Med..

[25] Corbí-Santamaría P., Gil-Calvo M., Herrero-Molleda A., García-López J., Boullosa D., García-Tormo J.V. (2025). The Impact of Advanced Footwear Technology on the Performance and Running Biomechanics of Mountain Runners. Appl. Sci..

[26] Maloney S.J. (2018). Review of the Badminton Lunge and Specific Training Considerations. Strength Cond. J..

[27] Nagano Y., Sasaki S., Higashihara A., Ichikawa H. (2020). Movements with greater trunk accelerations and their properties during badminton games. Sports Biomech..

[28] Wylde M.J., Lee M.B.C., Yong L.C., Callaway A.J. (2018). Reliability and validity of GPS-embedded accelerometers for the measurement of badminton specific player load. J. Trainol..

[29] Encarnación-Martínez A., Catalá-Vilaplana I., Berenguer-Vidal R., Sanchis-Sanchis R., Ochoa-Puig B., Pérez-Soriano P. (2021). Treadmill and Running Speed Effects on Acceleration Impacts: Curved Non-Motorized Treadmill vs. Conventional Motorized Treadmill. Int. J. Environ. Res. Public Health.

[30] Barrett S., Midgley A., Lovell R. (2014). PlayerLoad™: Reliability, convergent validity, and influence of unit position during treadmill running. Int. J. Sports Physiol. Perform..

[31] Bland J.M., Altman D.G. (1994). Statistics Notes: Correlation, regression, and repeated data. BMJ.

[32] Walter S.D., Eliasziw M., Donner A. (1998). Sample size and optimal designs for reliability studies. Stat. Med..

